# An Account of the Practice of One of the Physicians of the Westminster General Dispensary, and of the Western Dispensary, from the 20th of October, to the 20th of November, 1807

**Published:** 1807-12

**Authors:** Sam. Fothergill

**Affiliations:** Southampton-street


*An Account of the Practice of one of the Physicians of the Westminster General Dispensary, and of the Western Dispensary, from the 20th of October, to the 20th of November,* 1807.

| | | | |
|---|---|---|---|
| ACUTE DISEASES. | | Gastrodynia - - | 3 |
| Catarrh - - | 5 | Colica Pictonum - | 2 |
| Acute Rheumatism - | 6 | Chronic Rheumatism | 7 |
| Synochus - - | 2 | Chronic Ophthalmia | 2 |
| Scarlatina Anginosa - | 4 | Sciatica and Lumbago | 3 |
| Measles - - | 6 | Cephalalgia - - | 4 |
| Small-pox - - | 8 | Paralysis - - | 2 |
| Quotidian - - | 1 | Dropsy - - - | 6 |
| Acute Diseases of Infants | 12 | Dysury - - | 3 |
| CHRONIC DISEASES. | | Scirrhous Liver - | 1 |
| Cough and Dyspnœa | 25 | Hydrocephalus Internus | 1 |
| Pulmonary Consumption | 4 | Worms - - | 3 |
| Hæmoptoe - | 4 | Tumid Abdomen - | 2 |
| Asthenia - - | 10 | Cutaneous Diseases - | 5 |
| Jaundice - - | 4 | Chlorosis - | 6 |
| Diarrhœa - - | 5 | Amenorrhœa - - | 4 |
| Dysentery - - | 2 | Dysmenorrhœa - | 2 |
| Pyrosis - - | 2 | Menorrhagia - - | 1 |
| Dyspepsia - - | 6 | Abortion - - | 2 |

Small-pox is still very general; and many children are also affected with measles and scarlatina. For some days past the weather has been cold, with rain, snow, and high winds. Catarrh, cough, and pulmonary affections, begin now to augment the practitioner's list. Some phthisical cases, which had lingered through the summer and autumn, have had their usual termination accelerated, and some new ones have occurred.

 I lately

I lately attended a very distressing case, where much obscurity prevailed as to the cause of the illness. William Farmer, a strong muscular man, whilst carrying a sack of flour, suddenly complained of general faintness, and pain and uneasiness in the stomach; persisting, however, in taking another sack, he sunk down under it, nearly insensible; in which state he was carried home, and immediately attended by a surgeon-apothecary, who attempted to bleed him, but no blood followed the puncture of the lancet. On the fifth day of his illness I saw him. He then complained, as he had done from the first, of great uneasiness in the stomach and abdomen; the latter appeared considerably distended, from the umbilicus to the pubis; and this, he stated, took place soon after his first being attacked, and had continued ever since. He had no use and little sense of feeling in the lower extremities, which were swelled from below the knee to the toes; and two or three large vesicles, filled with yellowish serum, had formed upon the feet. No evacuation from the bowels had occurred for two days; the urine passed from him without his being conscious of it, and was said, though it afterwards appeared not to be so, in considerable quantity. When any fluid passes off by dribbling, as in some cases of hæmorrhage, or as in the present instance, inexperienced persons are apt to be deceived as to the quantity; and unless a practitioner be on his guard, he is frequently mis-led.

In this case, at first, I trusted to the report given me; yet had doubts respecting the distention of the abdomen, which eventually proved to be owing to an overcharged bladder. The mind was unaffected, the countenance sunk and pallid, the skin cool, pulse low, and of natural quickness; a brown fur covered the tongue. A large dose of calomel and rhubarb was ordered, and afterwards camphorated mixture and volatile alkali. The next day I found the physic had operated well, but the stools were evacuated without consciousness; the urine was also said to flow more freely; he complained of uneasiness at the scrobiculus cordis; he had had some sleep, and had taken broth and gruel. A blister was applied to the chest; the mixture and opening powder were repeated. On the following day he thought himself something better, though he had been troubled with nausea, and vomited up some bile; the stools were still passed involuntarily, and had the appearance of green mud. The mixture was repeated, with the addition of cinchona.

On

On the following day, being the fifth of my attendance, a catheter was introduced by my friend Mr. Chevalier, and about two pints and a half of urine, of natural appearance, were drawn off; the abdomen subsided, and great relief was immediately experienced.

Upon a very accurate examination of the spine, no injury was perceived; the legs and feet continued œdematous, and the insensibility of the lower parts remained as before. A long narrow blister was now applied the whole length of the spine; calomel and digitalis powder, of each one grain, with half a grain of opium, were directed to be taken three times a day; and the camphor mixture continued without the cinchona, which had not agreed with the stomach. He continued gradually sinking five days longer, when death terminated his sufferings. The urine was daily drawn off by the cathether; the stools, whether occasioned by physic or glysters, were passed involuntarily; he had no sense of pain; and his faculties continued unimpaired till nearly the last moments of existence. The only apparent effect produced by the calomel and digitalis, which were given to promote absorption, seemed to be increased action of the kidneys.

The body was opened by Mr. Chevalier, the day after death, when the process of putrefaction was already very forward; upon an incision being made across the parietes of the abdomen, the bowels instantly rose up, being exceedingly distended with gas, whilst the fœtor was intense. A quantity of serum, mixed with blood, was contained in the abdomen; all the viscera were sound, and the spine was uninjured. Upon separating the lumbar vertebræ, however, we found an extravasation of sanious matter; the substance of the nerves was soft and pulpy; they had not the usual white colour, and were easily torn. The Theca vertebralis appeared to have suffered a degree of inflammation; it was of a red colour, and had not its natural shining appearance. We, therefore, conjectured that a small blood-vessel, within the spine, had ruptured, and produced inflammation of the spinal marrow. The subject was very muscular, and great difficulty occurred in separating the lumbar vertebræ; during which operation, we were interrupted by the officiousness of the people of the house, and were thus prevented from more accurately tracing the diseased appearance of the medulla spinalis; but we did not suppose that it extended much beyond the first lumbar vertebræ. SAM. FOTHERGILL.

